# Brain-Derived Extracellular Vesicles in Health and Disease: A Methodological Perspective

**DOI:** 10.3390/ijms22031365

**Published:** 2021-01-29

**Authors:** Santra Brenna, Christoph Krisp, Hermann Clemens Altmeppen, Tim Magnus, Berta Puig

**Affiliations:** 1 Neurology Department, Experimental Research in Stroke and Inflammation (ERSI), University Medical Center Hamburg-Eppendorf, 20246 Hamburg, Germany; s.brenna@uke.de (S.B.); t.magnus@uke.de (T.M.); 2 Institute of Clinical Chemistry and Laboratory Medicine, Mass Spectrometric Proteomics University Medical Center Hamburg-Eppendorf, 20246 Hamburg, Germany; c.krisp@uke.de; 3 Institute of Neuropathology, University Medical Center Hamburg-Eppendorf, 20246 Hamburg, Germany; h.altmeppen@uke.de

**Keywords:** extracellular vesicles, BDEVs, brain, isolation protocol, sucrose gradient, mass spectrometry, central nervous system, proteomics, intercellular communication

## Abstract

Extracellular vesicles (EVs) are double membrane structures released by presumably all cell types that transport and deliver lipids, proteins, and genetic material to near or distant recipient cells, thereby affecting their phenotype. The basic knowledge of their functions in healthy and diseased brain is still murky and many questions about their biology are unsolved. In neurological diseases, EVs are regarded as attractive biomarkers and as therapeutic tools due to their ability to cross the blood–brain barrier (BBB). EVs have been successfully isolated from conditioned media of primary brain cells and cerebrospinal fluid (CSF), but protocols allowing for the direct study of pathophysiological events mediated or influenced by EVs isolated from brain have only recently been published. This review aims to give a brief overview of the current knowledge of EVs’ functions in the central nervous system (CNS) and the current protocols to isolate brain-derived EVs (BDEVs) used in different publications. By comparing the proteomic analysis of some of these publications, we also assess the influence of the isolation method on the protein content of BDEVs.

## 1. Introduction

### 1.1. Brief History of EVs

Since the 1960s, several studies reported evidence of the existence of extracellular vesicles-shaped membranous structures present in different tissues and organisms [1,2,3,4,5]. In 1967, Wolf et al. described minute lipid particles derived from platelets recovered after ultracentrifugation from serum and plasma, which showed “platelet-like” activity, thus confirming earlier observations made by Chargaff and West in 1946 [6,7]. They initially named this lipidic material “platelet dust”, and later changed it to microparticles. The term “extracellular vesicle” (EV) was first used in 1971 by Aaronson et al. to describe the secreted membranous structures they observed in *Ochronomas Danica*, the golden alga [8]. These EVs of different sizes were visualized using electron microscopy with and without fixation, excluding the possibility of just being an artifact. In the late 1970s, Ronquist and colleagues described extracellular vesicles (later named “prostasomes” [9]) with ATPase activity, secreted from prostate epithelial cells that functionally affected sperm cells [10]. In 1981, Trams and colleagues observed that shed “microvesicles” harvested from conditioned media of glioblastoma cell lines had a special membrane composition originating from certain plasma membrane microdomains [11]. These shed vesicles also induced an effect in recipient cells, a fact that had already been observed in cancer cells by others [12]. They proposed the term “exosomes” (as opposed to intracellular endosomes) for this type of shed extracellular vesicles [11]. In 1983, by studying the maturation of reticulocytes to erythrocytes, two papers published in parallel demonstrated that the elimination of the transferrin receptor was mediated by the extracellular release of vesicles originating from the endocytic compartment by fusion of multivesicular bodies (MVB) with the plasma membrane. The term “exosome” was later used for these types of vesicles (i.e., intraluminal vesicles, ILVs, released to the extracellular space) [13,14,15,16].

However, at that point, there was rather broad scepticism about specific EV functions, and exosomes were widely regarded as garbage bags produced by cells to discard obsolete, superfluous proteins [17]. In 1996, Raposo and colleagues, in a seminal paper, could show that antigen-presenting exosomes derived from B lymphocytes were capable of specifically stimulating T cells, bestowing functionality to exosomes [18]. Further work showed that the release of functional exosomes was a general mechanism for several types of cells, related or not to the immune response, such as dendritic cells [19], mast cells [20], platelets [21], and intestinal epithelial cells [22], among others [23]. As the interest for exosomes was growing, they were starting to be more thoroughly characterized [24] and differentiated from other types of released EVs such as apoptotic blebs [25]. As already observed by Trams et al. (although at that point named exosomes), vesicles shed from the plasma membrane in a regulated process were also recognised as a communication tool and named microvesicles, ectosomes, shed vesicles, and microparticles [26,27,28,29,30]. Another important milestone in the research of EVs as a means of intercellular communication was the proof that they contain genetic material that can be transferred to and translated within the recipient cell [31,32,33,34].

Related to neurodegenerative diseases, several amyloidogenic and pathogenic proteins such as amyloid β (Aβ), prion protein, tau, and α-synuclein utilize EVs for spreading throughout the brain, thus contributing to disease progression [35,36].

The interest for EVs has grown exponentially over the last years and different protocols for their isolation, characterization, and, more recently, for EVs extraction from tissues such as brain have been published. Despite many advances, current protocols and protein markers used for their characterization cannot specifically differentiate between exosomes (of endosomal origin) and ectosomes/microvesicles (shed from the plasma membrane). The International Society of Extracellular Vesicles (ISEV, founded in 2011) has since 2012 published position papers, with the arduous task of standardizing the nomenclature, protocols, and techniques for their characterization [37,38,39] and increasing experimental reproducibility. At present, if no specific proof of the origin of exosomes/ectosomes is available, the agreement is to name them EVs. They can be further grouped by sizes (i.e., small EVs (≤200 nm) or medium/large EVs (≥200 nm)), by their density, or by their biochemical composition [37].

The aim of this review is to give an overview of the studies published so far that isolated BDEVs and to analyze different protocols used to purify them, highlighting their major differences. Finally, we also compare published proteomic data obtained with different isolation protocols from mouse and human tissue to assess the influence of the isolation procedure on the analysis of the BDEVs content.

### 1.2. EVs in Central Nervous System (CNS) Physiology

It has been proven that all CNS cells release EVs, which are involved in numerous physiological and pathological processes [39,40,41,42,43]. In steady-state conditions, EVs from human-induced pluripotent stem cells (hPSC)-derived neurons increase neurogenesis, cell proliferation, and neuronal differentiation when incubated with human primary neurons. Similarly, EVs isolated from rat neuronal primary cultures and injected into the lateral ventricle of postnatal day 4 (P4) mice, lead to hippocampal neurogenesis, highlighting the importance of EVs in the development of neuronal circuits [44]. In neurons, the release of EVs carrying the α-amino-3-hydroxy-5-methyl-4-isoxazolepropionic acid (AMPA) receptor subunit GluR2 is regulated by calcium influx and glutamatergic synaptic activity, suggesting the involvement of EVs in synaptic transmission [45]. Glutamate can also stimulate vesicles release from oligodendrocytes [46], and these EVs are then taken up by neurons as support in stress conditions [47]. Moreover, EVs released by oligodendrocytes regulate myelin sheath formation in close coordination with neurons [48]. EVs released from astrocytes can regulate dendritic complexity in neurons via miR-26a-5p [49] and, conversely, EVs of neuronal origin containing miRNA 124a are taken up by astrocytes, leading to increased expression of excitatory amino acid transporter 2 (EAAT2) [50]. EVs released from microglia, on the other hand, can modulate neuronal activity in different ways: by stimulating the synaptic activity via enhanced sphingolipid metabolism [51], and by inhibiting the γ-aminobutyric acid (GABA)-ergic transmission via signaling of the endocannabinoid N-arachidonoylethanolamine (AEA) [52]. Moreover, the platelet-derived growth factor-BB (PDGF-BB)/PDGF receptor beta (PDGFRβ) signaling stimulates the release of EVs from pericytes carrying growth factors implicated in neuroprotection [42]. Lastly, EVs from brain endothelial cells promote oligodendrocyte precursor cell survival, motility, and proliferation [53].

### 1.3. EVs in CNS Pathologies

In pathological conditions, such as inflammation, extracellular astrocyte-derived ATP binds to the purinergic P2X7 receptor (P2X7R), activating microglia that then massively release EVs carrying IL-1β, thus further propagating the inflammatory response [40]. However, EVs released from ATP-stimulated microglia also contain a unique set of proteins that in turn impact astrocyte activation, showing then a protective role towards neurons [41]. Inflammatory microglia deliver EVs-associated microRNA 146-a-5p to neurons that negatively influence spine and synaptic density and strength [42]. As observed for microglia, astrocytes also increase EVs release when ATP activates the P2X7 receptors, and this process is associated with the release of the proinflammatory cytokine IL-1β [43].

Furthermore, it has been shown that EVs are involved in neurodegenerative diseases such as Alzheimer’s disease (AD), Parkinson’s disease (PD), and Creutzfeldt-Jakob disease (CJD). These diseases are characterized by the aggregation, deposition, and spread of specific misfolded proteins in particular regions of the brain: Aβ and hyperphosphorylated Tau for AD, α-synuclein for PD, and the pathogenic form of the prion protein (PrP^Sc^) for CJD [44]. For AD, it has been shown that EVs isolated from neuronal cells contain not only Aβ [45] but also the full-length amyloid precursor protein (APP, from which the Aβ fragment is generated by two subsequent proteolytic cleavages) and other APP-derived proteolytic fragments [46]. Moreover, one of the typical neuropathological features of AD, the extracellular amyloid plaques, are enriched in the exosomal marker protein Alix [45], pointing to a possible role for EVs in plaque formation. Tau is also released in association with EVs [47], with consequences to the cerebral spread of Tau pathology [48,49,50]. Cells overexpressing cytoplasmic α-synuclein release EVs containing this protein in a calcium-dependent manner and are toxic to primary neurons [47,51,52]. Lastly, both the cellular form of the prion protein (PrP^C^) and its infectious counterpart (PrP^Sc^), the key pathological molecule underlying all transmissible prion diseases in humans and animals, are found on EVs purified from prion-infected neuronal cell lines [53] and are capable to transmit the toxic prion conformation to other cells in culture [54]. However, it is still not clear if, in humans (where neurodegenerative diseases last for years), EVs are relevant disease propagators or rather represent a failed mechanism of clearing misfolded proteins, such as Aβ [55], or whether both aspects hold true to some degree [35]. Of note, EVs in the context of neurodegenerative diseases have also been extensively studied and discussed as potential biomarkers [36,56,57].

In other neurological conditions, such as ischemic stroke (IS) and traumatic brain injury (TBI), where initial localized damage (either by the blockage of a main brain artery or by a blow, bump, jolt or a penetrating object to the head) is followed by neuroinflammation, breakage of the blood–brain barrier (BBB), and infiltration of peripheral immune cells, the role of EVs in the disease outcome is much more complex (reviewed in [58,59,60,61,62]). In this context, it seems clear that EVs detected in CSF, plasma or blood could be valuable biomarkers of disease prognosis [63,64]. Moreover, treatment with EVs derived from mesenchymal stem cells (MSCs) shows promising results in improving the neurological outcome in animal models of stroke [65], and TBI, through a not-yet well-defined mechanism. Thus, therapeutical treatment with EVs is foreseeable in these acquired neurological disorders [66,67,68,69].

In a mouse model for Multiple Sclerosis (MS), an autoimmune disease characterized by demyelination and axonal injury in the CNS, myeloid microvesicles are significantly increased in the CSF compared to controls, and these EVs are capable to spread inflammatory signals both in vitro and in vivo [70]. Moreover, myeloid exosomes are increased in the CSF of patients with relapsing-remitting MS (RRMS) in comparison to healthy controls [70]. Plasma levels of endothelial EVs are increased in MS patients during the clinical relapse phase compared to the remission phase, pointing out a possible role for endothelial EVs as disease state biomarkers [71].

CNS-EVs in brain tumors have been intensively studied (reviewed in [72]). Cell lines of glioblastoma (GBM), an aggressive tumor of glial origin, secrete EVs with high immunogenic potential in mice and humans [73,74]. Serum EVs from patients suffering GBM are capable to polarize monocytes towards the anti-inflammatory phenotype M2, enhancing tumor growth in vitro [74]. Remarkably, astrocytes in the brain tumor microenvironment (TME) release EVs containing miR-19a, downregulating the tumor suppressor PTEN in tumor metastatic cells, thus contributing to their growth. When these cells exit the brain TME, PTEN function is restored [75].

Most of the above-mentioned studies were performed under in vitro conditions, or by isolating EVs from fluids. They have greatly helped to characterize the EV content and to the understanding of basic CNS-EVs functions. However, there is a clear need to retrieve and analyse EVs directly from brain tissue, to have a better picture of the whole physiological and pathological processes, including all cellular players. In 2012 the first paper isolating EVs from brain was published [76]. In the following years, modifications of this protocol and completely new protocols isolating BDEVs from both, healthy and diseased conditions, have been reported [49,77,78,79,80,81,82]. In the next paragraphs, we will analyse similarities and differences between the isolation methods and, for the sake of conciseness, we will only focus on protocols used for EVs isolation from brain. We will also re-examine and compare published proteomic analyses obtained by different isolation methods, to evaluate their efficiency and comparability in retrieving EVs from brain tissue.

## 2. BDEVs: Comparison of Current Protocols

As mentioned above, for over three decades EVs have been successfully isolated from cell culture media and body fluids (e.g., CSF, blood, urine, sperm, breast milk) [83]. Different protocols for EVs isolation from body fluids or conditioned media have been established, such as ultracentrifugation, immunoprecipitation, ultracentrifugation, size exclusion chromatography, or filtration-based concentration among others [84,85,86,87]. Notably, body fluids are generally more viscous than culture media as they contain numerous non-EV structures, such as lipidic components in plasma and serum, fat-containing vesicles in milk, and surfactant in bronchoalveolar lavage. For each type of body fluid, specific precautions have to be taken into account, as all of these non-EVs structures might be isolated together with EVs and interfere with the analyses [39,83]. An even more challenging procedure is to isolate EVs from complex tissues. To liberate the EVs from the extracellular matrix (ECM), the frozen or fresh tissue first must suffer an initial mechanical disruption (i.e., the tissue being cut into small pieces), generally followed by enzymatic digestion to disrupt the network of glycosaminoglycans, proteoglycans, glycoproteins, and fibrous proteins that compose the ECM. In the first protocol published in 2012 [76] and in later variations of this protocol [82,88], the enzyme of choice was papain, a cysteine protease found in papaya and often used, e.g., to prepare primary neuronal cultures. Another enzyme widely used is collagenase, which breaks the collagen peptide bonds of the ECM [77,78,79,81,89]. During this disruption procedure, the creation of artifacts, such as synaptosome-like vesicles formation, membrane damage, or contamination with intracellular vesicles, seems unavoidable, and therefore, apart from several rounds of centrifugation, further purification steps, such as membrane pore filtration (e.g., using a 0.2 µm filter) and density gradients, are applied. The final washed pellet from the fractions generated through the density gradient is enriched in BDEVs. Variations of this protocol include the enzymatic dissociation of the tissue together with automatized disruption [90].

The whole procedure is very time consuming and difficult to automate. Therefore, other protocols have also been optimized for BDEVs isolation such as size exclusion chromatography (SEC) [81], avoiding the density gradient step, or precipitation with organic solvents methods such as the PROSPR method, which avoids enzymatic digestion, ultracentrifugation, and density gradients ultracentrifugation [80]. Even though PROSPR has already been used to isolate EVs from plasma [91,92], to purify BDEVs it so far was the chosen and published method by one group only [80].

To evaluate the comparability of BDEVs isolated with different protocols, we have compared available proteomics data from different studies. For mouse brain, we have compared a paper recently published from our lab [78], a paper from Silverman et al. [93], and the mouse data published by Gallart-Palau et al. [80]. For human BDEVs, we have compared the proteomic data of Vella et al. [77], Huang et al. [81], and the human data from the paper of Gallart-Palau et al. [80].

**Figure 1:**
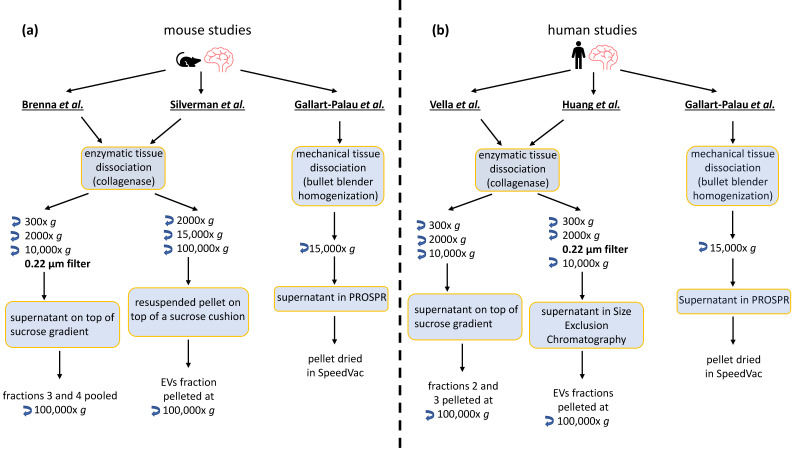
Summarizing scheme comparing protocols used for the proteomics analysis of BDEVs. (**a**) Schematic workflow of the protocols used in the mouse studies. (**b**) Schematic workflow of the protocols used in human studies.

Figure 1 shows the major steps of the protocols used in these studies, but for in-depth details, we of course suggest the reader refers to the original papers. Except for Gallart-Palau et al., all these studies included short tissue slicing and incubation with collagenase type III for not longer than 20 min at 37°. In the study from Gallart-Palau et al., they used a mechanical approach, consisting of a bullet blender homogenizer with metallic beads to disrupt the tissue.

After the dissociation step, low speed centrifugations (300× *g* and 2000× *g*), to clear cells, tissue fragments, and other debris are performed in Silverman et al., Huang et al., Vella et al., and Brenna et al. protocols, as depicted in Figure 1. Higher-speed centrifugations (10,000× *g* and 15,000× *g*) were used in all considered studies to further discard debris and/or larger vesicles. An additional filtration step (0.22 µm filter) is introduced either before the 10,000× *g* centrifugation (in Huang et al.) or after this centrifugation step (in Brenna et al.) to eliminate large EVs (≥200 nm). In the latter study, it was assessed that this filtration step was indeed discriminating between different EV populations as the filtered preparation was specifically enriched in different proteins (e.g., ribosomal proteins) compared to the unfiltered, as revealed by mass spectrometry [78].

In the protocols of Brenna et al. and Vella et al. the 10,000× *g* supernatant (filtered or not) is then overlayed on top of a sucrose gradient, centrifuged at 180,000× *g*, and designated fractions containing EVs were collected. Differently, in Silverman et al. the 10,000× *g* supernatant is first pelleted at 100,000× *g*, then resuspended, placed on top of a sucrose cushion, and centrifuged at 150,000× *g*. In the study of Huang et al., the supernatant collected after the 10,000× *g* centrifugation is further processed by SEC and the EVs are collected in specific eluate fractions. Gallart-Palau et al. isolate EVs with a solvent-based precipitation method coupled with low-speed centrifugation (PRotein Organic Solvent Precipitation, PROSPR), and the supernatant containing the EVs is dried in a SpeedVac.

Except for Gallart-Palau et al., all the other studies (EVs collected either from the sucrose gradient or from SEC) lastly pelleted the EVs at 100,000× *g* for further analyses.

## 3. Mass Spectrometry Analysis-Based Comparison between Different BDEVs Isolation Protocols

### 3.1. Methods

Mouse BDEVs proteomic data: as most raw data were not publicly available, the protein identification lists provided in the publications were used for our comparison. For the data sets of Gallart-Palau et al. and Silverman et al. the protein sequence database searches were performed with a combination of reviewed and non-reviewed protein sequence databases and, to better compare the studies, gene names were used and converted to Uniprot-reviewed protein accessions (www.uniprot.org). Proteins that were identified in at least one of the replicates per study were used for the analysis.

Human BDEVs proteomic data: Mass spectrometry raw data were either downloaded from the specified online repository or requested from the authors. In the case of Vella et al., the already processed protein list provided in the original publication was used.

Raw data were re-processed using the Andromeda algorithm in MaxQuant 1.6.3.4 (Max Planck Institute for Biochemistry, Martinsried, Germany) setting with carbamidomethylation of cysteines as a fixed modification and the oxidation of methionine as a variable modification. A reviewed human protein sequence database downloaded from Uniprot (EMBL, released in September 2020, 20,387 sequence entries) was used. Proteins with a protein and peptide false discovery rate of <0.01 percent were accepted as being present in the data set.

The representative Uniprot accession for each protein group was taken and Venn diagrams were generated using Venny 2.1 (https://bioinfogp.cnb.csic.es/tools/venny/index.html).

Cellular Component enrichment analysis based on Gene Ontology (GOCC) was performed using DAVID Bioinformatics Resources (DOI: 10.1038/nprot.2008.211). Ranking based on enrichment significance (*p*-value) was used. For comparison, the fifteen most significant enrichments across all data sets were used.

### 3.2. Results

**Figure 2:**
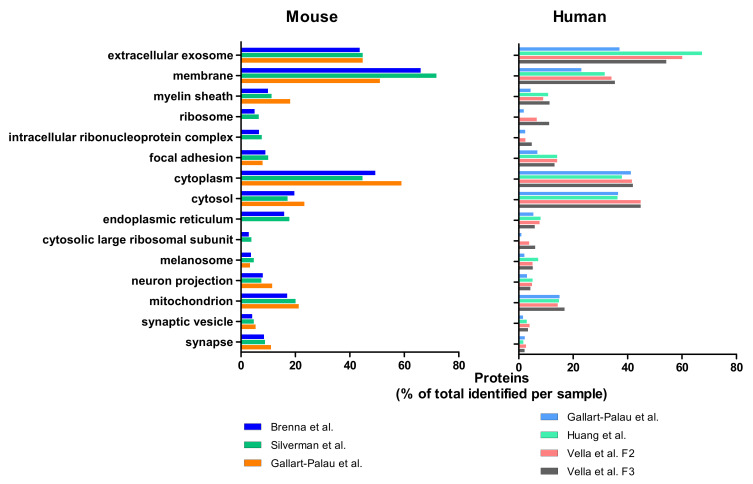
Bar charts of the 15 most enriched GO Cellular Components (GOCCs) in the mouse and human studies. The graph shows the top 15 GOCC detected with DAVID in the mouse studies (on the left) and the human studies (on the right).

**Figure 3:**
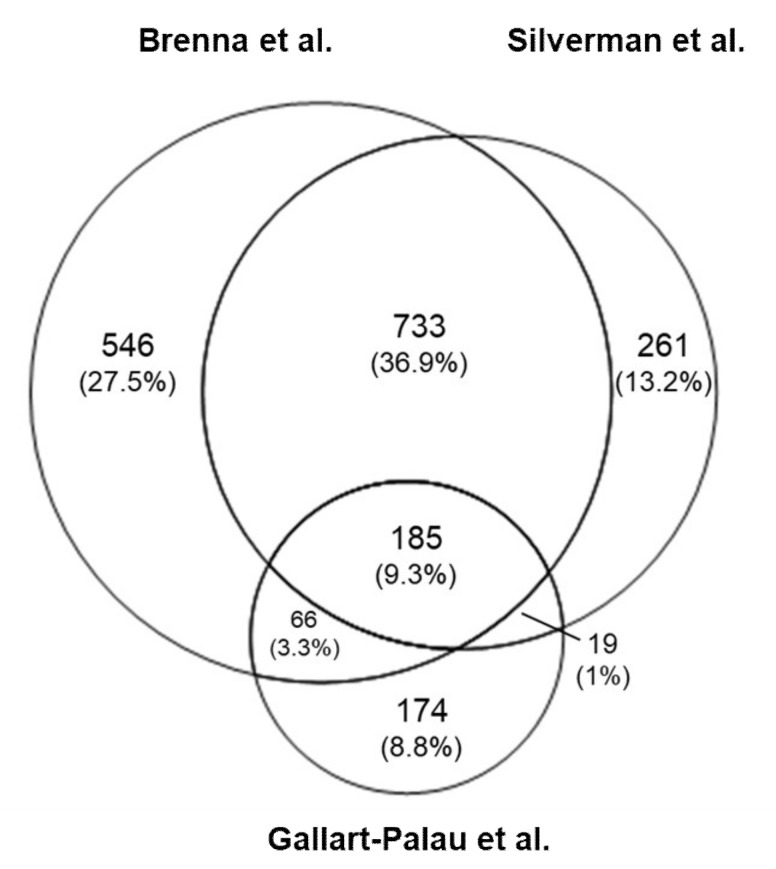
Venn diagram of all proteins detected in mouse studies. The data set from Brenna et al. detected 27.5% of proteins as unique, sharing 36.9% of proteins with the data set from Silverman et al. and 3.3% with Gallart-Palau et al. Silverman et al. detected 13.2% of proteins as unique, sharing with Gallart-Palau et al. 1% of proteins. Gallart-Palau et al. present 8.8% of proteins as unique. 9.3% of all proteins are shared among all three studies.

Regarding mouse BDEVs, Silverman et al. identified 1191 proteins in BDEVs, Gallart-Palau et al. found 444 proteins, and Brenna et al. 1518 proteins (Table 1). To better compare such different studies, we decided to not consider the absolute number of proteins detected in each study but their specific GO Cellular Component enrichments (GOCC) using the DAVID program. As depicted in Figure 2, the top 15 GOCC enrichments of the three data sets identified with DAVID and ranked by *p*-value include the GO term “extracellular exosome” (GO:0070062), highly enriched (over 40%) in all three mouse studies. Another largely represented GO term is “membrane” (GO:0016020) accounting for around 70% of proteins in Brenna et al. and Silverman et al., and 50% for Gallart-Palau et al. “Cytoplasm” (GO:0005737) accounts for 50% of the identified proteins in Brenna et al., around 45% in Silvermann et al., and almost 60% in Gallart-Palau et al. The terms “cytosol” (GO:0005829) and “mitochondrion” (GO:0005739) are around 20% of the identified proteins, whereas “focal adhesion” (GO:0005925) and “synapse” (GO:0045202) accounts for almost 10% of the proteins in all three studies. Even though all mouse data sets share similarities, the study of Gallart-Palau et al. shows a total absence of ribosomal components (i.e., “ribosome” (GO:0005840) and “intracellular ribonucleoprotein complex” (GO:0030529)) which in the other two studies account for around 5–7% of the identified proteins. A similar absence is observed for “endoplasmic reticulum” (GO:0005783), accounting for almost 20% in the Silverman et al. and Brenna et al. studies. On the other hand, the presence of “myelin sheath” (GO:0043209) is increased in the Gallart-Palau et al. study, accounting for more than 20% of the identified proteins. The differences are noticeable in the Venn diagram (Figure 3), where Silverman et al. and Brenna et al. share 36.9% of the detected proteins while the PROSPR study shares with them roughly 9.3%.

Regarding human BDEVS, Huang et al. identified 714 proteins, Gallart-Palau et al. 3056 proteins, while Vella et al. detected 1144 proteins in Fraction 2 and 815 in Fraction 3 (Table 1). For the latter study, we here analyze the two fractions most enriched with EV markers as described in the paper [77].

**Figure 4:**
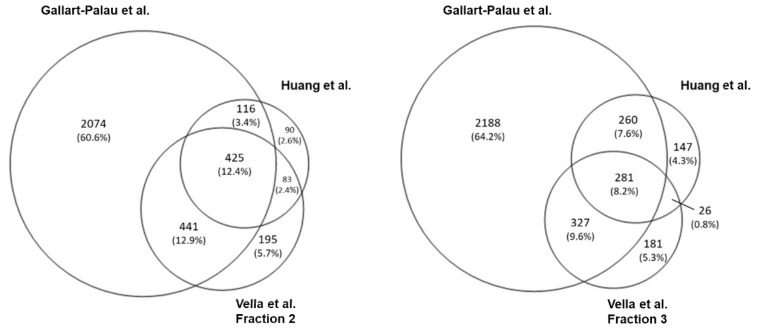
Venn diagram of all proteins detected in human studies. On the left, the Venn diagram for human studies including Vella et al. fraction 2. On the right, including Vella et al. fraction 3. Gallart-Palau et al. detected 60.6% of proteins as unique, sharing 12.9% of proteins with the data set of fraction 2 from Vella et al., and 3.4% with Huang et al. The latter detected 2.6% of proteins as unique while sharing with Vella’s fraction 2 the 2.4% of detected proteins. Fraction 2 from Vella et al. accounts the 5.7% of proteins as unique. 12.4% of all proteins are shared among the three studies. When considering Vella’s fraction 3, Gallart-Palau et al. detected 64.2% of proteins as unique, sharing 9.6% of proteins with the data set of fraction 3 from Vella et al., and 7.6% with Huang et al. The latter detected 4.3% of proteins as unique, sharing with Vella et al. fraction 3 0.8% of proteins detected. Fraction 3 from Vella et al. presents 5.3% of proteins as unique. 8.2% of all proteins are shared among the three studies.

As shown in Figure 2, in all three human studies, coinciding with the mouse studies, the most enriched GO term is “extracellular exosome”, accounting for nearly 70% identified proteins in Huang et al., 60% in Vella et al. (Fraction 2), and for 54% in Fraction 3, while in Gallart-Palau et al., this term accounts for 37% of the identified proteins. The GO term “membrane” is also highly enriched in all three studies, accounting for 38% (F2) and 37% (F3) in Vella et al., 35% in Huang et al., and around 23% in Gallart-Palau et al. “Cytoplasm” accounts for almost 40% of all the proteins in all three studies, and the same is observed for the term “cytosol” with 45% of the proteins identified falling on this term in F2 and F3 of Vella et al., and around 37% in Huang et al. and Gallart-Palau et al. Additionally, in all the human data sets “mitochondrion” is around 15% and “endoplasmic reticulum” between 5 and 8%. The GO term “focal adhesion” accounts for 7% in the PROSPR study and 14% of the total identified proteins in all the other human studies. Conspicuously, the human data of Gallart-Palau and colleagues, contrary to their own mouse study, retrieves proteins related to the terms “ribosome”, “intracellular ribonucleoprotein complex” and “cytosolic large ribosomal subunit”. In this case, the data set from Huang et al. does not identify proteins for these terms. The Venn diagram (Figure 4) shows that the number of identified proteins shared by Gallart-Palau et al. and Vella et al. are higher than in Huang et al. compared either to Vella et al. and Gallart-Palau et al. This is in contrast to the mouse studies where the PROSPR method of Gallart-Palau et al. was the least similar to the other two studies.

Overall, the graph in Figure 2 shows that the terms “exosome”, “membrane”, “cytoplasm” and “cytosol” followed by “mitochondrion” are the most enriched GO terms in the mouse and human proteomic studies compared herein, regardless of the method, implying a real enrichment of BDEVs in all of them. Nevertheless, we could observe some differences between the methods. Of note, it must be considered that all studies included in this review had different aims in terms of protein identification and quantification, and thus, they used different mass spectrometric strategies such as fractionation and proteome analysis applications. Moreover, different generations of mass spectrometers were used: Orbitrap Elite (Thermo Scientific) in Gallart-Palau et al., Orbitrap Fusion (Thermo Scientific in Brenna et al., Q Exactive Plus (Thermo Scientific) in Vella et al., Q Exactive HF (Thermo Scientific) in Huang et al. and Impact II Q-TOF (Bruker) in Silverman et al. Important to consider is also the overall quality of the tissue sample, in terms of post-mortem time and storage conditions, especially for the human brains [39].

However, by using the same MS strategy, the PROSPR method of Gallart-Palau et al. detected the highest number of proteins in the human brain, but the lowest in the mouse brain. It is hard to say if the method is indeed more effective in human brain than in mouse samples, as several variables can account for this discrepancy. For example, the amount of tissue used for the extraction was differing very much among the two experiments (40 mg of mouse brain tissue versus 150 mg of human brain tissue, as reported in the publication), thus introducing a potential source of variability. This could also be an explanation for why the authors did not find enrichment for ribosomal proteins in mouse brain but human. Still, as Huang et al. already pointed out in their study, the same method applied to human, macaque, and mouse tissue produces different results and yields. As the authors indicate, this could also be a consequence of either inter-species differences in tissue fragility or the processing of different brain areas with a different cellular composition [81]. Another possible source of variability can be the exact brain area used, especially in human studies (parietal cortex in Huang et al. versus frontal cortex in Vella et al. versus temporal lobe in Gallart-Palau et al.).

Brenna et al., Silverman et al., and Vella et al. found ribosomal-related proteins in the mass spectrometry analysis, whereas, as discussed, the PROSPR method only found these in humans, and in Huang et al. they were not detected at all. The first three studies share major steps in the EVs isolation protocols, suggesting that density gradients isolate BDEVs with similar characteristics and/or contaminants. Thus, the current methods used for BDEVs isolation show that some proteins are differentially enriched, or diverse unique proteins are identified, when the isolation is made by SEC or by chemical separation such as PROSPR, compared with density gradients and ultracentrifugation methods. However, as mentioned above, the amounts of tissue used in the studies were different, and the amount of protein evaluated in MS was not always reported, making any comparison difficult when certain proteins are not identified.

More recently, gradients using iodixanol with upwards floatation (instead of overlaying the sample) have been implemented in the isolation of EVs [79,94] which can help to minimize contamination and to yield the highest purity for BDEVs (our unpublished observations).

Finally, we would like to mention that, in the papers reviewed herein, the enzyme of choice was collagenase. However, papain has also been very successfully applied in several BDEVs isolation protocols [76,95,96]. From our own experience (unpublished observations) and that of others [97], when using papain it is important to consider the accuracy in incubation time and proper inhibition of the enzyme.

## 4. Future Perspectives

The study of BDEVs can significantly help to understand complex and multicellular physiological and pathological processes in the brain, which is simply not possible in in vitro studies.

Since the publication of the first protocol for isolation of BDEVs [76], the number of publications on this topic is increasing, with the first group of those publications reviewed in reference [98]. Since then, regarding neurodegenerative diseases such as AD, it has been shown that during the preclinical stage the expression of MHC class I markers in BDEVs is significantly upregulated [99] and that EVs isolated from murine brain are enriched with C-terminal fragments of APP (APP-CTFs), actively produced on the vesicles [76,100]. Furthermore, brain-derived EVs isolated both from early AD subjects and BCAS (bilateral common carotid stenosis) mice carry proteins involved in hypoxia such as EFEMP1, downstream activator of HIFs [101], highlighting the role of EVs in the hypoperfusion in human dementias. In a mouse model of PD, the inhibition of glucocerebrosidase (GCase) activity increased the amounts of BDEVs-associated α-synuclein oligomers [102]. Furthermore, another study showed that, when injected into mouse brains, EVs isolated from patients with Lewy Body Disease (LBD) were capable to induce α-synuclein aggregation [103]. In our lab, we recently demonstrated in mice that, under physiological conditions, microglia are the main source of small EVs (sEVs; <200 nm) in the brain sEV pool and that 24 h after experimental stroke, astrocyte-derived sEVs are significantly increased. Moreover, in the same study, we were able to describe a role for the prion protein (PrP^C^) and its proteolytically truncated C1 fragment in the uptake of sEVs by recipient cells [78]. Additionally, a recent study demonstrated enrichment of TDP-43 C-terminal fragments in BDEVs isolated from the motor cortex of ALS patients [104]. Lastly, it has been shown that EVs from different organs (e.g., brain, lung, heart) have specific markers, specifically synaptophysin (SYP) for brain-derived EVs, which are enriched also in other synaptic membrane proteins and receptors [105].

All in all, BDEVs are a particularly important tool for studying EVs-mediated intercellular communication in the brain in steady-state and relevant alterations in various disease conditions. It is difficult to draw clear conclusions on whether one isolation technique is better than another, at least by comparing proteomic analysis as we did here. All methods included in this review can successfully isolate BDEVs and differences in the proteomic analysis seem to be more dependent on other aspects, such as the amount and area of tissue used for isolation, and, for the proteomic analysis, the mass spectrometric strategy and analysis, and the employed mass spectrometer itself. As the field advances very rapidly, and as highlighted in this review, there is an obvious need for exact reporting standards (e.g., protein amounts, tissue regions) to make studies more comparable with reliable and reproducible results.

## Figures and Tables

**Table 1:** Total protein IDs identified in human and mouse studies. Note that there is a slight difference in protein numbers published in the referred papers and in Table 1. This is due to our strategy to use gene names and convert them to Uniprot-reviewed accessions as described in Material and Methods.

Human		Mouse	
Study	Proteins	Study	Proteins
Gallart-Palau et al.	3056	Silverman et al.	1191
Huang et al.	714	Gallart-Palau et al.	444
Vella et al. F2	1144	Brenna et al.	1518
Vella et al. F3	815		

## Data Availability

Not applicable.
